# Meningeal lymphatics as a therapeutic target for neurodegenerative disorders

**DOI:** 10.1186/s40035-025-00528-y

**Published:** 2025-12-09

**Authors:** Yijia Feng, Shengya Wang, Huwei Xia, Xinyi Jiang, Mingming Wu, Sipei Pan, Weihong Song

**Affiliations:** 1 Center for Geriatric Medicine, Key Laboratory of Alzheimer’s Disease of Zhejiang Province, The First Affiliated Hospital, Institute of Aging, Wenzhou Medical University, Wenzhou, 325027 China; 2 Oujiang Laboratory (Zhejiang Lab for Regenerative Medicine, Vision and Brain Health), Wenzhou Medical University, Wenzhou, 325035 China

**Keywords:** Meningeal, Lymphatic, Vessels, Immune, Neurodegenerative disorders, Alzheimer’s disease

## Abstract

Advancements in visualization methods have brought the meningeal lymphatic system (MLS) into the spotlight. The meningeal lymphatic vessels (mLVs) play a vital role in draining cerebrospinal fluid and immune cells, acting as a central hub for immune surveillance in the brain. Age-related morphological and functional declines of mLVs suggest their involvement in the pathogenesis of neurodegenerative disorders (NDDs). In this article, we summarize key discoveries about the MLS over the past decade, highlight the neuro-immune crosstalk in the meninges, and discuss the role of mLVs in both brain homeostasis and neurodegeneration. As a critical regulator of brain function and a potential therapeutic target, the MLS offers a promising avenue for the diagnosis and treatment of NDDs, particularly Alzheimer’s Disease.

## Introduction

Lymphatic drainage in the brain has been well studied. However, the brain was long perceived with a feature of absence of lymphatic vessels [1–4]. In 2015, two independent teams discovered the existence of dural lymphatic vessels, a breakthrough that has challenged the concept of the brain as an immune-privileged organ [5, 6]. The immune privilege concept emerged from early transplantation experiments showing that autologous tissues or sarcomas transplanted into the brain could survive without triggering significant immune rejection, unlike transplants in other regions, such as subcutaneous tissue [7, 8]. The rejections are primarily mediated by adaptive immune processes. The traditional understanding of brain borders, the impermeability of the blood–brain barrier (BBB) to immune cells, the presumed absence of a lymphatic system, and the belief that resting microglia cannot function as antigen-presenting cells (APCs) in the brain have shaped the immune privilege concept. In fact, this growth of foreign tissue in the brain occurs only when the graft is fully contained within brain tissue; while contact with the ventricles triggers a cellular reaction that leads to graft destruction [7]. The discovery of meningeal lymphatic vessels (mLVs) has prompted a re-evaluation of nervous and immune system interactions. These vessels drain cerebrospinal fluid (CSF) and immune cells into deep cervical lymph nodes (dCLNs), facilitating immune surveillance within the brain. Aplasia of mLVs impairs the clearance of macromolecules in the brain, significantly reducing drainage to the cervical lymph nodes (CLNs) [5, 6].

The pathophysiological role of mLVs was initially investigated in Alzheimer's disease (AD) [9]. Disruption of mLVs facilitates the accumulation of amyloid β (Aβ) protein in the brains of AD transgenic model mice. Impaired meningeal lymphatic function slows the perivascular influx of macromolecules into the brain, hinders their efflux from interstitial fluid (ISF), and induces cognitive deficits in AD mice [9]. In comparison to their drainage function, the role of mLVs as a critical neuroimmune interface that facilitates the connection between the central nervous system (CNS) and peripheral immune responses is often overlooked. Dysfunction of the mLVs may lead to immune dysregulation within the brain, thereby contributing to the pathogenesis of various neurodegenerative disorders (NDDs). For instance, in a mouse model of multiple sclerosis (MS), ablation of meningeal lymphatics mitigates pathological manifestations and reduces the inflammatory response of brain-reactive T cells [10]. Additionally, mLVs can be targeted to enhance lymphatic drainage of brain tumor antigens, promoting immune surveillance and protecting against CNS tumors [11, 12]. Recent research on mLVs has allowed for the visualization of these structures in humans [13, 14], paving the way for deeper exploration of their function and development of therapeutic and interventional strategies for neurological disorders.

**Fig. 1:**
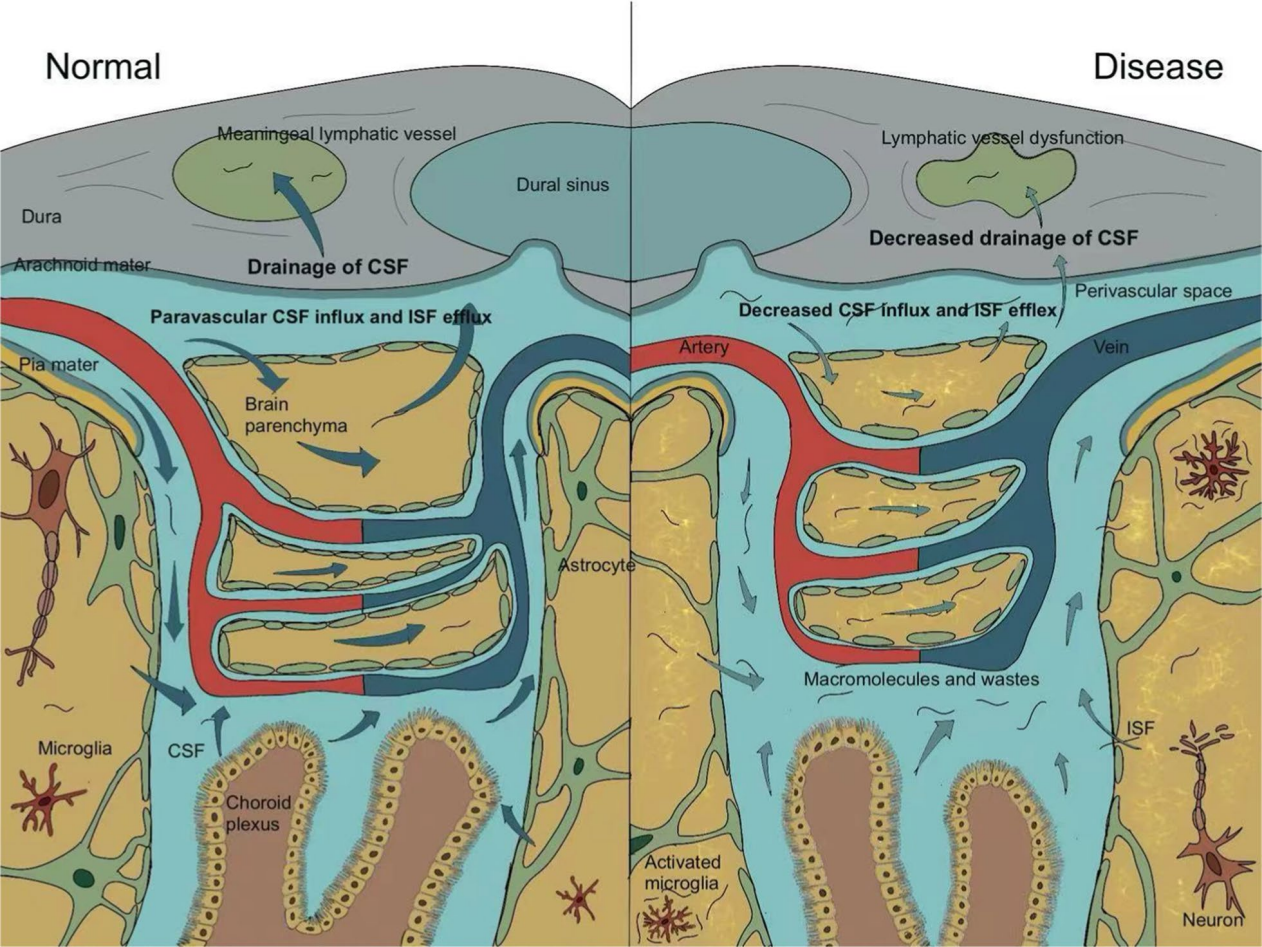
Meningeal lymphatic dysfunction hinders CSF drainage and decreases CSF–ISF exchange in the brain. CSF is produced by the choroid plexus and diffuses into the brain parenchyma through the paravascular spaces, where it exchanges with ISF. This process constitutes the glymphatic circulation, which supports the clearance of interstitial solutes. The mLVs then facilitate the drainage of CSF and ISF to the cervical lymph nodes. In pathological conditions, dysfunction of lymphatic vessels impairs CSF drainage, reduces its diffusion into the brain parenchyma, and disrupts ISF outflow. The loss of perivascular astrocytic endfeet further slows glymphatic clearance, potentially leading to neuronal damage and neuroinflammation

In addition to the blood circulation system, which delivers nutrients and removes metabolic waste, there exists a CSF circulation system that is essential for maintaining normal brain function. CSF is produced by the epithelial cells of the choroid plexus and circulates continuously throughout the subarachnoid space of the brain, facilitating functions such as the exchange with ISF. Both CSF and ISF are then expelled from the brain. Previous studies indicated that the CSF is absorbed into bloodstream through arachnoid granulation, thereby eliminating the possibility of immunological surveillance of this fluid. However, modern noninvasive imaging techniques that quantify tracer transport now demonstrate that the mLVs serve as another drainage pathway for CSF [13, 15]. CSF transports brain-derived antigens and immune cells to the vicinity of the dural sinuses, where they are captured by local APCs and subsequently migrate to draining lymph nodes through lymphatic vessels. This process enables patrolling T cells to recognize antigen-MHC complexes presented by APCs, completing their priming for immune surveillance [16, 17]. The glymphatic circulation allows CSF to enter the brain parenchyma via the periarterial space, then drain through the perivenous and perineuronal spaces, eventually exiting the brain through the MLS [18, 19]. Dysfunction of the meningeal lymphatic drainage disrupts the exchange between CSF and ISF, leading to the accumulation of metabolic waste or abnormal protein deposition in the brain, which further exacerbates brain injury in pathological conditions (Fig. 1).

In this article, we summarize the roles of meningeal lymphatic vasculature in the onset and progression of NDDs, and discuss current visual detection methods and the potential of mLVs as therapeutic targets for future interventions.

## Structure, composition and development of mLVs

The brain’s lymphatic system was first described over 200 years ago by an Italian anatomist and illustrator Paolo Mascagni, who provided a clear depiction of the mLVs and their physiological roles in the *Vasorum lymphaticorum corporis humani historia et ichnographia* (*History and Graphical Representation of the Lymphatic Vessels in the Human Body)* published in 1787 [20]. Despite this early discovery, the lack of evidence for mLVs in the brain at the time contributed to a long-standing but incorrect belief in its absence. Only recently have scientists begun to fully acknowledge and investigate the presence and roles of mLVs in the CNS.

Immunofluorescence staining of lymphoendothelial cell markers in tissue samples is essential for visualizing mLVs. Key markers include LYVE1 (lymphatic vessel endothelial hyaluronan receptor 1) [21], PDPN (podoplanin) [22], vascular endothelial growth factor receptor 3 (VEGFR3) [23], transcription factor PROX1 (prospero homeobox protein 1) [24, 25] and CCL21 (chemokine (C–C motif) ligand 21) [26]. Selective double or multiple immunostainings have become a more effective strategy to accurately visualize these vessels [14]. Furthermore, magnetic resonance imaging (MRI) techniques such as 3D-rendering of subtraction MRI images [14] and real-time vessel-wall MRI following systemic injection of gadobutrol [13], also facilitate this visualization.

The meninges, the protective membrane surrounding the CNS, are composed of the outer dura mater and the inner leptomeninges. The leptomeninges include the arachnoid and pia mater, while functional lymphatic vessels are located within the outermost layer—the dura mater [5, 27]. The meningeal lymphatic vasculature has been confirmed in both humans and animals, with their precise anatomical locations gradually being elucidated [5, 6, 13, 14, 25, 28–31]. These lymphatic vessels develop postnatally, following distinct growth paths on the rostral and caudal sides of the skull, sprouting in characteristic patterns along veins, arteries, cranial nerves, and spinal nerves [32]. Vascular endothelial growth factor-C (VEGF-C), primarily expressed in vascular smooth muscle cells and also present in the pituitary and pineal glands, is essential for mLV development [33, 34]. A recent report has shown that VEGF-C, as a key mediator, regulates glial-mediated lymphatic development and contributes to neural activity–dependent lymphatic growth [35]. In the meningeal lymphatic network, capillary lymphatics converge into pre-collect vessels, which is a universal anatomical structure containing specialized valves to prevent lymph backflow. The pre-collect lymphatics then exit the cranial brain to collect lymphatic vessels, which are wrapped by smooth muscle cells [5, 13, 29, 32, 36].

**Fig. 2:**
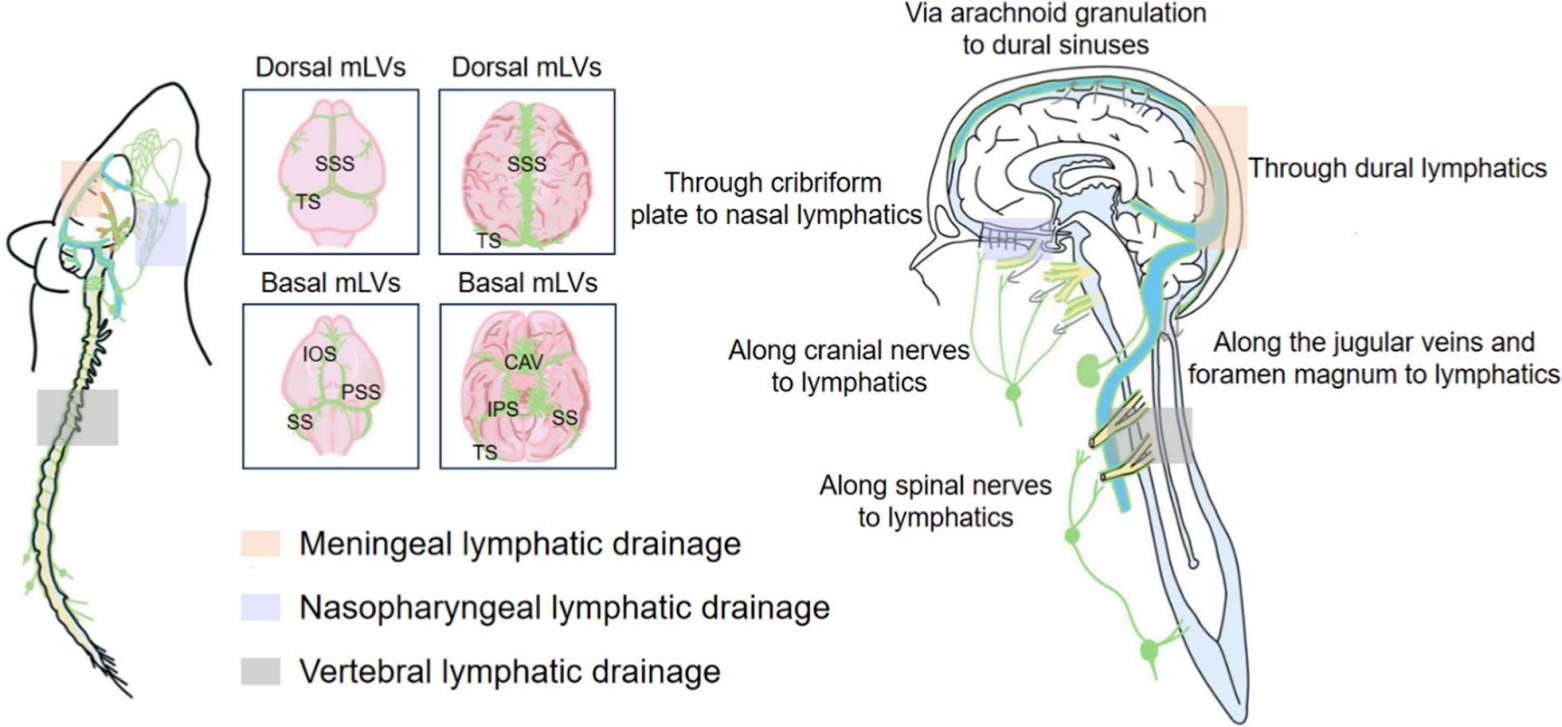
Overview of MLS anatomy and associated CSF efflux pathways. Schematic illustration of mLVs (green) in mice and humans with corresponding anatomical regions. Human MRI data and murine tracer studies reveal species-specific differences in the distribution of dorsal and ventral dural lymphatic vessels. These variations likely reflect methodological differences and anatomical divergence, although in both species mLVs consistently track alongside blood vessels and cranial nerves. The major CSF lymphatic drainage pathways—meningeal, nasopharyngeal, and vertebral—are delineated in distinct colors at their corresponding sites in both species. In addition to these three well-characterized routes, CSF is also drained along blood vessels and nerves, which are marked with gray arrows in the human brain

Studies on mLVs have primarily focused on the dorsal and basal regions [5, 6, 14, 28, 29]. In the mouse brain, dorsal mLVs are clustered within the dura along the superior sagittal sinus (SSS) and transverse sinuses (TS), whereas basal mLVs extend along the petrosquamous sinuses (PSS), sigmoid sinuses (SS) and inferior olfactory sinus (IOS) [5, 6, 29] (Fig. 2). Dorsal mLVs are small in diameter, with largely discontinuous vascular structures, often lacking lymphatic valves and exhibiting a continuously sealed, zipper-like junctional pattern indicative of immature morphology. In contrast, basal mLVs are larger, with numerous blunt ends protruding capillary branches, composed of typical oak leaf-shaped lymphatic endothelial cells (LECs) and lymphatic valves. These vessels display a prominent, loosely sealed, button-like junctional pattern in the LECs [5, 29]. Damage to meningeal lymphatics increases susceptibility to stress-induced depression and anxiety-like behavior in mice, affecting females but not males [37]. However, the mechanisms by which sex regulates mLVs remain unclear.

In the human brain, dorsal mLVs are visible around nearly all dural venous-parasagittal structures and are attached to the base of the skull. Basal mLVs are found not only in the anterior cranial fossa but also around the cranial foramina and cranial nerves [28]. The density of dural lymphatics is greater around the venous sinuses than in more lateral areas of the dura [14]. They also exhibit a wide range of maximum transverse diameters, likely due to the fact that many are collapsed [14, 31]. Although the volume and thickness of mLVs are significantly larger in men than in women, the overall lymphatic throughput is similar in both sexes[13, 28]. Notably, CSF and ISF drainage channels exist in both the dorsal and ventral systems, and changes in body position may have an impact on the balance or the ratio of CSF-ISF drainage in humans [14, 28].

In both species, mLVs consistently track alongside blood vessels and cranial nerves. In mice, substantial evidence demonstrates their functional significance, encompassing the menigeal, nasopharyngeal, and vertebral lymphatic drainage pathways [9, 38, 39]. In humans, MRI has progressively identified lymphatic vessels across multiple regions. Dorsal mLVs are located along the SSS and TS, whereas ventral pathways primarily involve the cavernous sinus, inferior petrosal sinus, and SS [14, 28]. The associated CSF efflux route is also illustrated in Fig. 2.

## Lymphatic drainage pathway of CSF

CSF, produced by the choroid plexus, fills the brain’s ventricles and subarachnoid space [40]. Its molecular components are cleared through multiple pathways (Fig. 2), including transport into the dural sinuses via arachnoid granulations, drainage to cervical lymph nodes through nasal lymphatics, and, more recently, via the newly discovered mLVs, which have emerged as important routes for CSF clearance [5, 6, 38]. By injecting fluorescent dyes—fluorescein (i.v.), QDot655 (i.c.v.), and Alexa488-conjugated anti-Lyve-1 antibody (i.c.v.) into anesthetized adult mice [5], researchers demonstrated that whereas mLVs drain CSF at a slower flow rate, they do so in a similar direction as adjacent blood vessel. CSF drains into the CLNs via meningeal and nasopharyngeal lymphatic vessels [5, 6, 38, 41, 42]. While the primary drainage route of dCLNs remains under debate [5, 38], these two pathways clearly overlap at medial cervical lymphatic vessels. Further research is needed to compare between these pathways and their roles in various neurological disorders. Together with cranial routes, spinal lymphatic drainage provides an additional pathway that supports CNS homeostasis and warrants further investigation [39, 43].

Approximately 10% of CSF is derived from ISF within brain tissue. ISF is formed through perivascular exchange channels that transport fluids and solutes from the brain's interstitial space to the CSF [15]. This process depends on the glial water flux, primarily mediated by Aquaporin 4 (AQP4), and is a component of the glymphatic system [44, 45]. Tracers injected into the ISF have been shown to translocate into the CSF via glymphatic circulation, while simultaneously filling dural lymphatic vessels at the base of the skull [6]. This suggests that the glymphatic system serves as an upstream pathway for the meningeal lymphatic drainage, facilitating the clearance of CSF/ISF from the subarachnoid space into the mLVs [6, 46]. Specifically, meningeal lymphatics drain the majority of CSF/ISF into the dCLNs and partially into the superficial CLNs (sCLNs) [5, 6, 29, 47]. When the efferent lymphatic vessel of the dCLN was ligated, increased filling of the dural lymphatic vessels was observed [6]. Disruption of the meningeal lymphatic drainage further leads to reduced brain parenchymal perfusion and CSF drainage (Fig. 1).

Age is one of the factors affecting the drainage function of mLVs [15, 28, 29, 46]. Old mice show increased basal mLVs size, highly branched and proliferative phenotypes, degeneration of the dorsal mLV branches, and reduced coverage of the SSS by the dorsal mLVs compared to young mice [15, 29]. Besides, basal mLVs from old mice display dysmorphic distribution of type IV collagen and have fewer lymphatic valves compared to those from young mice [29]. However, in the human brain, the thickness of mLVs increases in the ventral system with age, with even more pronounced thickening in the dorsal system [28]. This may be related to morphological differences between mouse and human brains, as well as body position during imaging [28]. Aging compromises the integrity of mLVs and CSF drainage, resulting in reduced clearance and atrophy of the CLNs [15, 28, 46, 48]. Notably, age-related changes in mLVs are organ-specific and do not differ by sex [29].

In addition, the mechanical force generated by lymphatic flow is essential for the postnatal development of mature and functional meningeal lymphatics [48]. plcγ-2^−/−^ mice, which exhibit reduced lymph flow, display impaired structural remodeling and maturation of mLVs, as well as decreased macromolecule uptake and transport. This suggests that the formation of mature mLVs is positively correlated with increased drainage of macromolecules from the CNS to the dCLNs, and that functional mLVs depend on adequate lymphatic flow [48].

## Neuro-immune crosstalk in meninges

The brain was considered an immune-privileged organ, separated from peripheral immune responses. However, numerous studies have shown that the CNS is closely linked to the immune system. Some immune-related cytokines can modulate synaptic plasticity, support neuron survival, promote memory, and mitigate social behavior deficits [49–51], while other cytokines negatively affect brain function. For example, IFN-γ inhibits neural stem cell regeneration and is associated with social behavior impairment [52–54]. IL-17a is essential for maternal immune activation and has been linked to abnormal behavior in offspring [55]. IL-6 expression in microglia can mediate inhibitory synaptic phenotypes, leading to behavioral alterations [56]. Interestingly, the brain can even store and recall specific immune responses [57]. Neurotransmitters and neuropeptides influence immune cells [58–61]. Recent findings further highlight the importance of immune activity at brain borders, its role in neural function, and its involvement in neurological disorders. In the dural sinus hub, the immune system can sample CNS antigens, drain immune cells, reactivate T cells locally, secrete inflammatory factors, and influence microglial activation, thereby contributing to nervous system activities [16, 17, 62, 63].

**Fig. 3:**
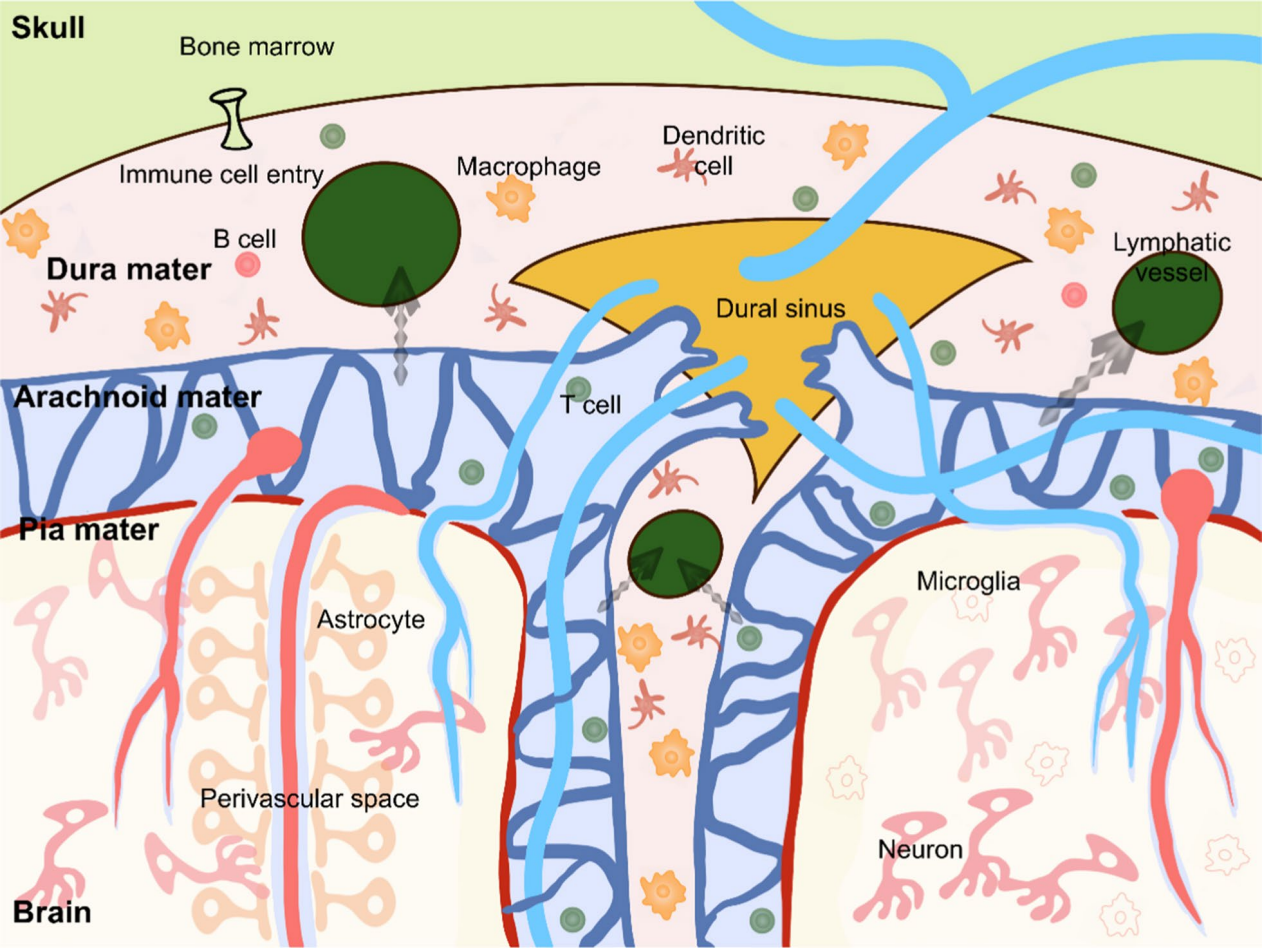
Neuro-immune crosstalk in the meningeal hub. The meninges serve as a supportive niche for various immune cell populations. Brain-derived antigens and immune cells accumulate around the dural sinuses, an area rich in stroma and abundant in APCs. Draining lymphatic vessels enable antigen presentation and the priming of immune cells both locally and in the cervical lymph nodes. The bone marrow within the skull is characterized by specialized channels that facilitate the entry of immune cells into dural tissue, providing critical immunological support when needed. The CSF harbors memory T cells that patrol the CNS. These cells, together with brain-derived antigens, are transported to the dural sinuses and then conveyed to the cervical lymph nodes through mLVs, facilitating immune surveillance within the brain

Meningeal-derived signals play an active role in regulating various brain functions [54, 62, 64]. Unlike other brain borders, such as perivascular spaces (PVS) or choroid plexus, the meninges offer distinct advantages in neuro-immune crosstalk (Fig. 3). First, the meninges house a rich reservoir of immune cells. These diverse immune cells reside within the meninges, performing specific immune functions and contributing to various disease processes [16]. Meningeal γδT cells express high levels of the chemokine receptor CXCR6, and their production of IL-17a is implicated in the regulation of behavioral outcomes in experimental models of autism [62]. Type 2 innate lymphoid cells, another immune cell type residing in the meninges, can be activated through an IL-33-dependent pathway following spinal cord injury. Blocking this signaling by knocking out IL-33R promotes injury recovery [65]. Interestingly, the bone marrow of the skull and vertebrae contains specialized channels independent of blood circulation, that allow immune cells to migrate to the meninges. Monocytes, neutrophils, and B cells found in the mouse meninges originate from the skull and vertebrae, entering the meninges through these unique vascular pathways [66–70]. In both homeostasis and disease states, these bone marrow-derived immune cells strategically infiltrate the meninges, interact with resident immune cells, and carry out distinct regulatory functions. Second, cytokines released by meningeal immune cells influence neurons, regulating synaptic plasticity and short-term memory, while also affecting social behavior and exploratory activities [54, 62, 63, 71]. The meninges host a diverse population of immune cells, making it crucial to investigate the functions of these various cell types and their roles in brain disorders. Third, mLVs drain brain-derived antigens and T cells, playing a key role in immune surveillance [5, 6, 29]. Contrary to the traditional belief that the CSF flows directly from the venous sinuses into the bloodstream, studies have found that when fluorescent tracers are injected into the CSF, they appear in the lymphatic circulation before reaching the bloodstream [15, 72]. mLVs drain CSF and immune components into the CLNs, facilitating antigen presentation and immune cell activation, thereby enhancing the neuro-immune communication.

The CSF surrounds and permeates the brain parenchyma within the skull, maintaining constant contact with various brain borders. In healthy individuals, most immune cells in the CSF are memory phenotype T cells, with presence of only a few dendritic cells and B cells [73–75]. The meninges, in contrast, contain a richer population of immune cells, primarily concentrated around the dural venous sinuses. This region is home to numerous APCs, including dendritic cells and MHC class II-positive macrophages, as well as T cells, B cells, plasma cells, neutrophils and others [16, 76–79]. CSF from the subarachnoid space can flow to the dura mater, where antigens originating from the brain and CSF are recognized and processed by dural APCs. These cells then present the antigens to patrolling T cells, facilitating immune surveillance [16, 80, 81].

Dural macrophages are predominantly bone marrow-derived mononuclear phagocytes and play an antigen-presenting role in neuroinflammation and neurodegeneration, while also secreting cytokines that influence neural activity [79, 82, 83]. Perivascular and leptomeningeal macrophages, located near the CNS, have been shown to act as cellular regulators of CSF flow dynamics [76]. Dendritic cells are primarily located at vascular-rich borders, such as the choroid plexus and dura mater. However, their roles in facilitating T cell antigen presentation and participating in immune regulation during disease states require further investigation [84, 85]. CSF acts as a courier, detecting homeostasis and danger signals, prompting immune niches at brain borders to interact with the nervous system. The emitted immune signals can potentially support brain function repair or exacerbate disease progression. Meningeal adaptive immune activity is prominent and has been shown to play a significant role in multiple neurological diseases [81, 86, 87]. For instance, meningeal T cells play a role in limiting AD pathology, maintaining normal cognitive function, and mediating dopaminergic neurotoxicity [88–90]. The dural stromal cells also express chemokines, such as CXCL12 and CXCL16, which promote the chemotactic recruitment of lymphoid and myeloid cells. Similar to the choroid plexus stroma, the dural stroma is rich in extracellular matrix and ligands, providing a habitat for tissue-resident memory T cells [16, 66]. Additionally, this site facilitates B cell negative selection as they encounter CNS antigens [91]. Lymphocytes patrolling the CNS are essential for defending against foreign pathogens. They also secrete pro-inflammatory cytokines and chemotactic molecules, regulating brain function [92, 93]. Interestingly, non-immune cells in the dura, such as mural cells—including pericytes and smooth muscle cells, interact with macrophages to regulate CNS immune surveillance. Through physical contact, mural cells transfer cytoplasmic components to macrophages in the dura, which is essential for inhibiting antigen-dependent T helper cell activation and TH17 cell differentiation [94].

## Roles of mLVs in NDDs

The mLVs undergo increased morphological and functional impairments with age [9, 95], suggesting their involvement in the pathogenesis of age-related NDDs. Moreover, mLV dysfunction disrupts normal brain homeostasis and is linked to other neurological disorders associated with CSF drainage and neuroinflammation. These findings underscore the urgent need for the development of visual detection methods and therapeutic strategies targeting the mLV system.

### The mLVs in AD

AD is the most common neurodegenerative disorder, characterized by the formation of extracellular Aβ plaques and intracellular tau protein aggregates [96–98]. These pathological changes contribute to neuronal damage, neuroinflammation, progressive cognitive decline, and memory loss [99–102]. Positron emission tomography imaging to detect Aβ and tau proteins in the brain provides essential support for clinical diagnosis and research [103]. Reducing the accumulation and promoting the clearance of Aβ and tau proteins are primary strategies for preventing and treating AD [102, 104–114].

**Fig. 4:**
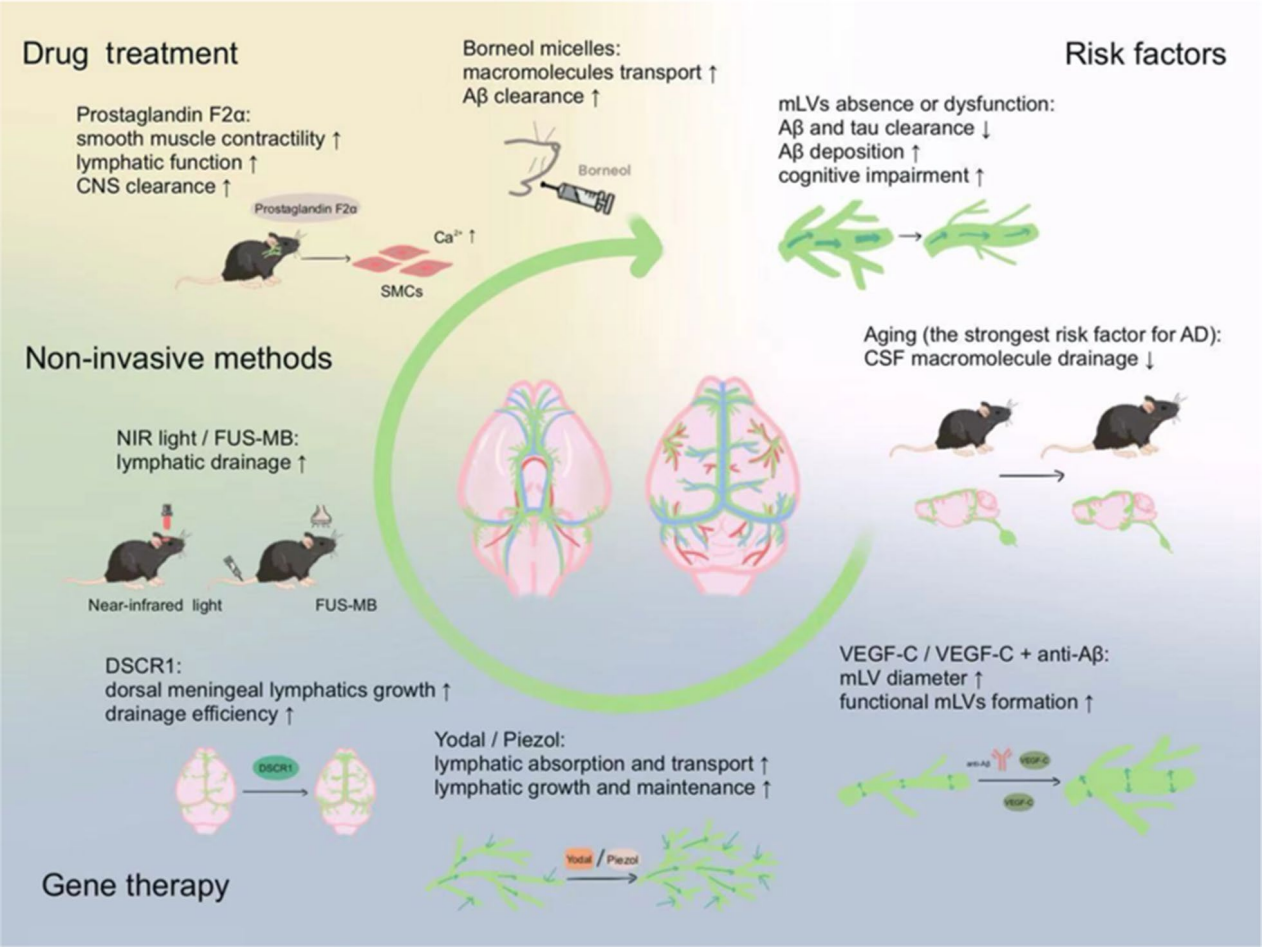
The meningeal lymphatic system (MLS) is closely linked to the development of AD. The center of the diagram depicts a network of lymphatic vessels in the dorsal and ventral meninges. Dysfunction in the MLS disrupts cerebrospinal fluid (CSF) drainage in mice, leading to delayed clearance of Aβ and tau proteins, increased Aβ accumulation, and impaired cognitive function. Aging, the strongest risk factor for AD, is closely associated with impaired function of the MLS. Current research on gene therapy (VEGF-C, Yoda1/Piezo1, DSCR1), non-invasive methods (FUS-MB, NIR light), and drug therapy (Prostaglandin F2α, Borneol) has demonstrated improvements in MLS. These approaches enhance drainage in the CSF and interstitial fluid, contributing to improved cognitive function and AD pathology

mLVs as a newly recognized pathway for CSF drainage, have been closely linked to AD [6, 9, 105, 115–117] (Fig. 4). Dysfunction of these vessels in 5×FAD mice reduces Aβ plaque clearance and impairs cognitive function. Additionally, in transgenic mice lacking dural lymphatic vessels, tau clearance from the brain is delayed [118]. VEGF-C is closely associated with the development and maturation of mLVs, which increases mLV diameter [5, 32], promotes the formation of functional mLVs (especially basal mLVs) [29, 31], and enhances the drainage of macromolecules from CSF and ISF, thereby contributing to improved cognitive function [9, 119]. Therapeutic delivery of VEGF-C improves the outcomes in 5×FAD mice treated with anti-Aβ passive immunotherapy [104]. Another study demonstrated that DSCR1 (Down syndrome critical region 1, also known as RCAN1, regulator of calcineurin 1) protects against AD pathology by promoting the growth of dorsal meningeal lymphatics, thereby improving the drainage efficiency [117]. Oral administration of small molecule compound borneol micelles can enhance the transport of macromolecules injected into the CNS via mLVs and accelerate the lymphatic clearance of Aβ in the brain. This effect is achieved by increasing the levels of FOXC2, VEGF-C, and LYVE-1 in the meninges to promote lymphangiogenesis, as well as reducing nitric oxide levels to stimulate lymphatic contraction [115]. In addition, it appears that Aβ clearance in the brain is impaired in the majority of patients with AD, regardless of whether there are changes in Aβ production [120–122]. Specifically, carriers of early-onset AD (EOAD)-associated presenilin mutations show increased Aβ production and decreased Aβ clearance [120, 123], while individuals with late-onset AD (LOAD) have stable Aβ production during aging, but their Aβ clearance is slowed [121, 122]. Therefore, the MLS is of considerable importance for clearance of abnormal protein aggregates.

A recent study shows that persistent atrophy or hyperplasia of mLVs induced by blocking or overexpressing VEGF-C in AD mice, affects CSF drainage but does not significantly alter the overall brain Aβ plaque burden [124]. This seems to contradict the common notion in most studies that disruption of mLVs leads to aggravated Aβ accumulation [9, 104, 105, 117]. This discrepancy may be related to different methods to target mLVs, although similar AD models were used in these studies. Greater Aβ accumulation was observed with verteporfin-mediated cranial photodynamic ablation and direct ligation of mLVs. In contrast, this accumulation was not seen with a single injection of AAV encoding the VEGF-C/VEGF-D trap [124]. How does the dysfunction of meningeal lymphatic drainage affect the pathology of AD? While direct evidence of meningeal lymphatic drainage of Aβ is lacking, mLVs may still influence AD pathology by affecting the brain's fluid exchange system, such as glymphatic circulation [9, 125], through existing or yet unknown clearance mechanisms. Moreover, the possibility that the meningeal immunity impacts the pathological process of AD, such as through decreased microglial response to Aβ clearance or impaired peripheral immunity, cannot be ruled out. In addition, factors regulating the VEGF-C pathway to promote mLV formation remain unclear. We speculate that other substances may synergistically contribute to the VEGF-C-mediated formation of mLVs [9, 104, 126]. Overall, the specific mechanisms by which the MLS mediates AD-related phenotypes require further investigation.

In recent years, numerous studies have examined the relationship between the glymphatic system and AD, highlighting the crucial role of CSF drainage in clearance of macromolecules, inflammatory mediators, and immune cells [6, 19, 105, 125, 127]. For example, deficiency of AQP4 that is essential for the glymphatic system, exacerbates tau hyperphosphorylation, inhibits tau clearance and contributes to neurodegeneration in the P301S mutant-tau transgenic mouse model [44, 128]. In addition, the loss of perivascular AQP4 localization is also associated with increased Aβ load [45, 127]. On the one hand, Aβ itself can disrupt glymphatic flow [129, 130], which, in turn, significantly inhibits both CSF inflow and ISF outflow via the glymphatic pathway. This severely impairs the drainage of Aβ and tau from CSF to the dCLNs [6, 105, 128], creating a vicious cycle that accelerates the pathogenesis of AD. As the downstream pathway for CSF drainage, proper functioning of the MLS is essential for efficient glymphatic circulation. Impairment of the meningeal lymphatics has been shown to hinder CSF entry into the brain parenchyma, disrupt ISF outflow, and contribute to cognitive impairment [9]. The two systems may synergistically contribute to the onset and progression of AD. However, further studies are needed to explore the mechanisms underlying their interaction, how macromolecules and immune cells drain from the CSF into mLVs, and their roles in disease development.

Given the critical roles of the glymphatic system and MLS in clearing abnormal protein aggregates from the brain [9, 104, 127, 128], enhancing lymphatic function and drainage to remove misfolded and toxic molecules may be beneficial for patients with AD. Extensive preclinical studies are needed to assess the feasibility of surgical interventions in lymphatic drainage as a treatment option, and their effectiveness for NDDs must be validated by well-designed clinical trials.

Aging, the strongest risk factor for AD [103, 131–134], is also associated with impaired functions of multiple clearance systems, exacerbating cognitive decline and Aβ pathology [125, 135]. Compared to young counterparts, changes in mLVs in old mice lead to reduced cerebral perfusion by CSF macromolecules [9, 29]. However, treatment with VEGF-C significantly improved meningeal lymphatic drainage of CSF macromolecules in aged mice [9]. In addition, aging is associated with impaired expression of AQP4 protein in the frontal cortex, and loss of perivascular AQP4 localization promotes the erroneous aggregation of Aβ in the aging brain [45]. As a downstream pathway for draining solutes and CSF, cervical lymphatic vessels show reduced contraction frequency and flow velocity in aged mice. Prostaglandin F2α treatment in aged mice restores the lymphatic function and improves clearance [95]. Additionally, the age-related decline of CCR7 expression in meningeal T cells is associated with an enhanced meningeal regulatory T cell (Treg) response, increased Aβ pathology, and cognitive decline. Treatment with an anti-CD25 antibody has shown potential in attenuating the Treg response and improving cognitive function in aged mice, further highlighting the meningeal lymphatic vasculature as a promising immunomodulatory target for therapeutic intervention [125].

In conclusion, mLV dysfunction is closely associated with aging. Dysregulation of mLVs contributes to the development and progression of AD. Targeting meningeal lymphatics could be a valid therapeutic avenue for AD.

### The mLVs in Parkinson’s disease (PD)

PD is a common neurodegenerative disorder in the elderly, characterized by motor symptoms such as tremor, rigidity and shuffling gait, along with non-motor symptoms like anosmia, sleep disturbances and autonomic dysfunction [136]. Abnormal aggregation of α-synuclein (α-syn) and degeneration of dopaminergic neurons are the primary pathological features of PD [137–139]. Recent studies suggest that the dural lymphatic system helps clear metabolites and pathological proteins [6], including α-syn.

Following ligation of the dCLNs in mutant α-syn A53T transgenic PD model mice, both intracellular and extracellular α-syn accumulation are increased in the substantia nigra, along with greater dopaminergic neuron loss and aggravation of behavioral deficits. Additionally, the entry of the fluorescent tracer TR-d3 into the brain parenchyma and the drainage function of dural lymphatic vessels are significantly reduced. This is accompanied by enhanced glial cell activation, elevated production of inflammatory cytokines, and worsened depolarization of AQP4 molecules (which are known to facilitate the drainage of water and pathological proteins) [140–143]. These findings suggest that impaired meningeal lymphatic drainage not only contributes to the pathology and motor impairments in a PD mouse model, but also disrupts the glymphatic system in the brain, further accelerating disease progression. In another study of dynamic contrast-enhanced magnetic resonance imaging (DCE-MRI) in PD patients, idiopathic PD showed less lymphatic drainage in the SSS and sigmoid sinus and significantly delayed dCLNs perfusion, compared with atypical PD patients and control individuals. Intracerebral injection of α-syn preformed fibrils in mice results in delayed meningeal lymphatic drainage, increased meningeal inflammation, and disrupted tight junctions between LECs. Moreover, blocking the mLV flow worsens motor and memory impairments and exacerbates α-syn pathology [144]. Thus, it is evident that mLVs play a crucial role in the pathology and progression of PD. However, no further studies have confirmed whether restoring damaged mLVs can improve PD pathology and behavior. Additionally, there is no direct evidence supporting that meningeal drainage affects the glymphatic circulation in the brain.

Similar as in AD patients, MRI data from 179 PD patients showed a significantly larger global PVS volume compared to controls [145]. PVS serves as a clearance pathway for the brain, allowing the influx of CSF to brain parenchyma and the efflux of ISF to the MLS. Changes in PVS volume indicate abnormal CSF circulation and drainage, which is closely associated with dysfunction of the glymphatic system and potentially the MLS [146–148]. Mice overexpressing human A53T-α-syn exhibit reduced AQP4 expression and impaired glymphatic circulation. Reduction or depolarization of AQP4 leads to glymphatic dysfunction, worsening PD pathology [149]. The MMP-9/β-DG pathway has been shown to regulate glymphatic function by modulating AQP4 polarization [150]. The glymphatic system activity is primarily concentrated during sleep and regulated by circadian rhythms [151, 152]. The absence of AQP4 in PD mice may explain the frequent sleep disorders experienced by PD patients. Enhancing glymphatic function offers potential for addressing PD pathology and may also help alleviate sleep disturbances.

Da Mesquita et al. used the photodynamic drug Visudyne (verteporfin for injection) to ablate mLVs by injecting it into the CSF. They found that meningeal lymphatic impairment slowed the entry of perivascular macromolecules into the brain parenchyma and disrupted the outflow of macromolecules from the brain's interstitial fluid, leading to significant cognitive impairment in mice [9]. In addition, CCR7 deficiency mimics the age-related changes in meningeal T cells, resulting in reduced glymphatic influx [125]. MRI scans of PD patients showed significant impairment in meningeal lymphatic drainage and enlarged PVS [144, 145], further suggesting that a functional crosstalk may exist between meningeal lymphatic outflow and glymphatic circulation. However, more data are needed to confirm the precise impact of meningeal lymphatic drainage on glymphatic function. Improving meningeal lymphatic drainage may enhance the exchange between CSF and ISF in the PVS, potentially mitigating pathological and behavioral deficits in PD.

### The mLVs in multiple sclerosis (MS)

MS is an autoimmune disorder characterized by immune cell infiltration into the brain and demyelination [153, 154]. This pathological process is commonly modeled in mice by immunizing them with myelin antigens, resulting in the development of experimental autoimmune encephalomyelitis (EAE) [155, 156]. The EAE mouse model is widely used to study the infiltration of autoimmune T cells into the brain and their attack on myelin proteins [81, 87]. However, the precise mechanisms by which autoimmune T cells acquire their encephalitogenic phenotype and trigger disease progression require further investigation. The existence of mLVs suggesting a significant connection between the CNS and the peripheral immune system, offers new insights and research directions.

mLVs are an important pathway for draining CSF macromolecules and immune cells [5]. Ablation of meningeal lymphatics reduces EAE pathology and dampens the inflammatory response of brain-reactive T cells. This process impairs the drainage of brain-derived antigens, limiting T cell entry into dCLNs in a CCR7-dependent manner and restricting T cell priming in the draining lymph nodes [10]. Ultrasound-guided harvesting of CLN cells in MS patients revealed the presence of myelin protein, providing a basis for T cell priming in these lymph nodes. In addition, surgical removal of lymph nodes leads to reduced disease severity in EAE models [157, 158]. Notably, earlier studies found that VEGF overexpression exacerbates the inflammatory response in EAE, potentially by promoting the migration of inflammatory cells to the lesion site [159].

Similar to meningeal lymphatics, nasal mucosa lymphatic vessels have been previously reported to be conduits for the drainage of CSF, as well as brain-derived antigens and immune cells [42, 160]. Lymphatic vessels near the cribriform plate drain CSF to the superficial CLNs (sCLNs), while mLVs drain CSF into both the sCLNs and dCLNs. Lymphangiogenesis and lymphatic vessel remodeling are commonly observed biological processes during inflammation [161]. In EAE mouse model, lymphatic vessels near the cribriform plate undergo lymphangiogenesis in a VEGF-C–VEGFR3-dependent manner. However, there are no morphological changes in the brain meningeal lymphatics [160]. The heterogeneity of these two sites of lymphatics still needs further investigation. Spinal lymphatics, predominantly situated in the epidural space above the dura mater, not only provide an additional CSF drainage route, but also act as an immune gatekeeper, as VEGF-C-induced lymphangiogenesis aggravates inflammation, T cell infiltration, and demyelination after spinal cord injury, suggesting a critical yet underexplored role in EAE pathogenesis [39, 43].

The discovery of meningeal lymphatic vasculature reveals a complete drainage pathway that allows CNS antigens to be sampled by the peripheral immune system. Under homeostatic conditions, antigen-activated T cells are not exposed to the CNS due to the BBB [162]. However, in the EAE mouse model, following peripheral subcutaneous injection of myelin protein, CNS-infiltrating encephalitogenic T cells attack myelin, leading to pathological demyelination in the brain. Given the intact structure of the BBB, how do activated immune cells progressively infiltrate the brain and cause inflammatory damage? Studies have shown that the adenosine A2a receptor in the choroid plexus mediates the early infiltration of peripheral T cells in EAE, facilitating the subsequent entry of a large number of inflammatory cells into the brain [163, 164]. The recently discovered meningeal lymphatics, like choroid plexus at the border of the brain, also contribute to CNS immune surveillance [5, 16, 93, 165]. It is believed that a similar molecular regulatory mechanism may exist in the meningeal lymphatic vasculature, playing a role in regulating disease onset and progression. Notably, in the early stages of EAE, invading antigen-specific T cells need to be reactivated by APCs at the border of the brain before they initiate neuroinflammation [87, 166]. The pathophysiology of APC–T cell interactions within the meninges is worth further investigations.

Reduced flow through mLVs along the SSS has been observed by DCE-MRI in neuromyelitis optica spectrum disorder (NMOSD) patients during acute attacks. The disease severity, as evaluated by the Expanded Disability Status Scale, correlates with the drainage function of mLVs-SSS [167]. This finding suggests that DCE-MRI could potentially predict acute NMOSD relapses by assessing mLV function. Future studies should aim to provide more objective MRI data from clinical patients to confirm whether meningeal lymphatic dysfunction occurs in MS. Additionally, further research is needed to determine whether enhancing meningeal lymphatic drainage offers therapeutic benefits in the early stages of the disease, and whether combining this approach with monoclonal antibody therapy could improve treatment outcomes for MS.

## Targeting mLVs for monitoring and therapy

The discovery of mLVs has fundamentally reshaped our understanding of the mechanisms underlying the clearance of CSF and ISF from the CNS [5, 6]. Previous studies have characterized the structure and function of rodent mLVs using transmission electron microscopy, histology, and fluorescent tracers [10, 168]. Recently, dual-contrast functional photoacoustic microscopy has been employed to achieve wide-field intravital imaging of the lymphatic system, encompassing both mLVs and glymphatic pathways, enabling the capture of dynamic drainage and clearance processes across the entire brain in mice [169]. However, there are substantial genetic and biological differences between humans and model animals. In rodents, molecular clearance from CSF occurs through drainage via the cribriform plate to the nasal mucosa [42], whereas human studies did not show significant contrast agent accumulation in the nasal turbinates [170]. Moreover, the timing of CSF tracer efflux from the brain also differs across species, reflecting distinct clearance dynamics in humans and mice [171, 172]. Therefore, developing effective methods to assess mLVs in humans is an urgent challenge that requires immediate attention.

To gain a comprehensive understanding of the pathophysiological role of mLVs in humans, non-invasive imaging is an indispensable first step. MRI is a preferred choice for visualization. High-resolution clinical MRI was first employed in 2017 to image mLVs in both humans and non-human primates. Utilizing T2 fluid-attenuated inversion recovery (FLAIR) and T1-weighted black-blood sequence, gadobutrol was found to enhance the visibility of these vessels, and subsequent pathological analysis confirmed the presence of a dural lymphatic network. This technique has been widely used in research on aging, NDDs, cerebral small vessel pathology, and brain tumors [46, 144, 173, 174]. Dynamic contrast-enhanced (DCE)-MRI can even be used to quantify mLV function, enabling its assessment in clinical applications [144]. By combining time-of-flight (TOF) angiography sequences, the direction of blood flow within intracranial vessels can be precisely determined without the use of exogenous contrast agents, thereby offering profound insights into the complex interactions between various vascular compartments. A study involving six healthy subjects revealed that the lymphatic flow in mLVs is oriented in the opposite direction to the venous flow in the SSS. A recent study using 2D interslice flow saturation MRI not only revealed the shape and distribution of mLVs, but also provided insights into their flow direction and velocity [175]. Another non-contrast sequence FLAIR has also been employed to assess mLV function, where the visibility of inferior frontal sulcal hyperintensity may serve as an indicator of clearance dysfunction of the mLVs [176]. This approach is based on the theory that metabolic waste products in the CSF, such as proteins and cellular debris, can increase signal intensity and cause hyperintensity on FLAIR imaging. Similarly, high FLAIR signals have been found around nearly all parasagittal dural venous structures, which seemed to be continuous with fluid pathways extending from the skull base to the dCLNs, indicating that mLVs may directly drain into the dCLNs [28]. 7 T-MRI enables more precise quantification of anatomical details and enhances tissue contrast, making it a potentially more sensitive method for mapping mLVs. In healthy individuals scanned at 7 T, the putative mLVs are predominantly localized around the SSS and cortical veins. The observed FLAIR signals and enhancement patterns suggest that these structures may be involved in the transport of protein-rich fluid [177]. MRI still faces major challenges, particularly the substantial mismatch between conventional voxel sizes in MRI (~ 1 mm) and the micrometer-scale diameters of mLVs (~ 10–100 µm) [178]. Nevertheless, current findings provide new evidence supporting the presence of intracranial mLVs. This could provide insights into new biomarkers for monitoring the progression of the disease and identifying novel therapeutic targets, providing crucial insights into how these vessels contribute to the pathogenesis of neurological disorders.

Currently, VEGF-C enhances mLV function, offering protective effects in various neurological disorders [11, 104, 179]. In Twist1^+/-^ mice, a model of craniosynostosis, impaired meningeal lymphatics and brain perfusion are observed. However, treatment with VEGF-C rescues these disease-associated phenotypes. Transplanting skull progenitor cells into the mutant mice promotes the growth and migration of LECs through VEGF-C secretion [180]. VEGF-C is also widely used in combination therapies to improve treatment outcomes. For instance, Da Mesquita et al. reported that VEGF-C delivery improved the outcome of 5 × FAD mice undergoing anti-Aβ passive immunotherapy by reducing Aβ deposits and other AD-related pathology, suggesting synergistic therapeutic potential of lymphangiogenic treatment and Aβ-immunotherapy [104]. Immune checkpoint inhibitors targeting the PD1 pathway have shown limited success in treating glioblastoma. However, Song et al. reported that transfection of an mRNA construct expressing VEGF-C improved the efficacy of immune checkpoint blockade therapy in a mouse model of glioblastoma by enhancing CD8^+^ T cell priming against brain tumors [11].

Piezo1 is a key regulator of lymphatic valve formation and fluid flow-induced lymphatic expansion. Transgenic Piezo1 expression or pharmacological activation enhances lymphatic regeneration. The Piezo1-mediated lymphangiogenesis has been shown to effectively suppress postsurgical lymphedema development [181, 182]. Yoda1 is a synthetic small molecule that acts as an agonist for both human and mouse Piezo1. It enhances Piezo1 activity in lymphatic vessels, promoting lymphatic sprouting and expansion [182, 183]. Choi et al. demonstrated that selective overexpression of Piezo1 in lymphatics or systemic administration of Yoda1 ameliorated brain lymphatic malformation and ventricular enlargement in mice with DS or hydrocephalus [184]. CSF appears to provide mechanical force that supports meningeal lymphatic growth and maintenance. Yoda1 treatment restores the reduced CSF flow in aged mice by improving brain-CSF circulation and enhancing lymphatic drainage of CSF to the dCLNs [185].

Since the mLVs are located on the surface of the skull, delivering light treatment to this area seems straightforward. These vessels have been implicated in various neurological disorders and represent potential therapeutic targets. A recent study demonstrated that a near-infrared light enhances lymphatic drainage, significantly improving cognitive function in aging mice. This treatment has also shown strong therapeutic effects in transgenic AD mice, reducing Aβ deposition and improving cognitive function and other AD-related phenotypes [168]. Another study reported that focused ultrasound treatment combined with microbubbles (FUS-MB) enhances the clearance of solute Aβ from the brain to the CSF and subsequently to the dCLNs. Ligation of the dCLNs exacerbates the formation of pathological plaque and memory impairment, while FUS-MB treatment causes improvement [186]. Furthermore, phototherapy applied to intraventricular hemorrhage in four-day-old male rats demonstrated a significant recovery effect, attributed to the enhancement of lymphatic drainage and clearance functions [187].

Flickering light stimulation is also being explored as a non-invasive therapeutic strategy for modulating neuropsychiatric disorders. Studies have shown that 40 Hz flickering light promotes sleep through the adenosine signaling pathway [188], and reduces Aβ deposition in the visual cortex of 5×FAD mice [189]. Combined auditory and visual stimulation at 40 Hz (multisensory gamma stimulation) significantly reduces amyloid plaques throughout neocortex and improves cognitive impairment in 5×FAD mice [190]. Further research found that this multisensory gamma stimulation promotes CSF influx and ISF efflux, facilitating Aβ clearance. The underlying mechanism involves increased arterial pulsatility and AQP4 polarization along astrocytic endfeet [191]. Loss of perivascular AQP4 localization has been shown in the aging human brain and is closely linked to AD pathology [45]. In AD mice, deletion of *Aqp4* slows perivascular CSF-ISF exchange and impairs Aβ clearance [143]. Interestingly, high-intensity interval training has also been shown to alleviate cognitive dysfunction and AD-related pathology by regulating astrocyte phenotype-associated AQP4 polarization [142]. It would be interesting to study whether flickering light stimulation or exercise can improve the meningeal lymphatic drainage and the phenotype of meningeal dysfunction-related diseases. A recent study has found that multisensory gamma stimulation leads to the dilation of mLVs, which may contribute to the clearance of Aβ pathology [191].

Non-invasive manipulations show promise in modulating the MLS [192]. However, the effectiveness of these approaches requires further validation under both normal and pathological conditions. In comparison to conventional intravenous injection, the mLV route enhances the transport of curcumin to the brain by about 20 times by delivering a natural killer cell membrane biomimetic nanocomplex [193]. This highlights the mLVs as a more efficient and straightforward route for targeted delivery of drugs to the brain. Currently, other therapeutic strategies to enhance meningeal lymphatic function are also being explored, including small molecule drugs and additional regulatory signals [38, 115]. Clinically, there is an urgent need to develop longitudinal MRI studies to assess the dynamics of human CSF and meningeal lymphatic drainage across various neurological diseases. It is also important to explore other imaging methods to more accurately assess the structure and function of mLVs, thereby supporting the development of new preventive, diagnostic, and therapeutic strategies for neurological disorders.

## Conclusion

The MLS plays a key role in clearing metabolic waste and abnormal protein deposits from the brain, while also contributing to immune surveillance by draining brain-derived antigens and immune cells within its unique immune environment. Several therapeutic strategies targeting the MLS have been proposed for neurological diseases (Table 1), although these approaches remain at the preclinical research stage. Future studies should further explore the similarities and differences of the MLS between animals and humans.

**Table 1:** Potential therapeutical strategies to modulate MLS for neurological diseases

Strategy	Applications in animal models	Mechanisms	Impact of the treatment	References
VEGF-C	Aging/AD	Increases mLVs diameter; Promotes the formation of functional mLVs	Facilitates amyloid-β clearance; alleviates cognitive deficits	[9, 104, 126]
	Tumor	Promotes priming of CD8 T cells, DC trafficking and CD8 T cell activation	Enhances radiotherapy efficacy; Potentiates checkpoint therapy	[11, 12, 194]
	TBI	Restores lymphatic function	Decreases neuroinflammation	[179]
	Stroke	Promotes lymphatic growth	Reduces stroke injury and ameliorates motor performances	[195, 196]
	JEV infection	Promotes expansion of functional mLVs	Alleviates infection-induced neurological damage	[119]
	HE	Promotes lymphangiogenesis	Decreases neuroinflammation and ameliorates motor dysfunction	[197]
Yoda1/Piezo1	DS/Hydrocephalus	Improves lymphatic absorption and transport	Decreases CSF accumulation and ventricular enlargement	[184]
	Aging/ Craniosynostosis	Promotes lymphatic growth and maintenance	Reduces intracranial pressure and improves CSF flow	[185]
Phototherapy	Aging/AD	Improves lymphatic drainage	Promotes pathological remission and cognitive enhancement	[168]
	IVH	Improves lymphatic drainage	Provides fast recovery after IVH	[187]
Prostaglandin F_2α_	Aging	Increases smooth muscle contractility	Restores lymphatic function	[95]
Borneol	AD	Promotes lymphangiogenesis	Accelerates the clearance of Aβ	[115]
FUS-MB	AD	Improves lymphatic drainage	Improves Aβ clearance	[186]
RCAN1	AD	Increases the coverage of dorsal mLVs	Diminishes Aβ pathology; Improves memory defects	[117]

TBI: traumatic brain injury; JEV: Japanese encephalitis virus; HE: hepatic encephalopathy; DS: Down syndrome; IVH: intraventricular hemorrhage; FUS-MB: focused ultrasound treatment in combination with microbubbles; RCAN1: regulator of calcineurin 1; VEGF-C: vascular endothelial growth factor-C

In addition, several avenues warrant further in-depth investigation, such as whether and how meningeal immune mechanisms contribute to the therapeutic effects of VEGF-C in AD; the long-term effects of mLV ablation if ablating meningeal lymphatics can reduce excessive inflammatory responses in MS; and whether early regulation of meningeal immunity could potentially slow disease progression. In addition, more studies are needed to identify factors that regulate meningeal lymphatic drainage and whether they are disease-specific. Lastly, imaging of meningeal lymphatics in larger populations is crucial for predicting or monitoring disease progression, as well as for identifying new diagnostic and therapeutic targets for NDDs.

## Data Availability

Available upon request.

## Abbreviations

NDDs: Neurodegenerative disorders
MLS: Meningeal lymphatic system
VEGF-C: Vascular endothelial growth factor-C
dCLNs: Deep cervical lymph nodes
LECs: Lymphatic endothelial cells
SSS: Superior sagittal sinus
TS: Transverse sinus
SS: Sigmoid sinus
PSS: Petrosquamous sinuses
IOS: Inferior olfactory sinus
Treg: Regulatory T cell
APCs: Antigen-presenting cells
AQP4: Aquaporin 4
PVS: Perivascular space
Aβ: Amyloid β protein
AD: Alzheimer’s Disease
EOAD: Early onset AD
LOAD: Late onset AD
BBB: Blood brain barrier
CLNs: Cervical lymph nodes
sCLNs: Superficial cervical lymph nodes
dCLNs: Deep cervical lymph nodes
CNS: Central nervous system
CSF: Cerebrospinal fluid
EAE: Experimental autoimmune encephalomyelitis
ISF: Interstitial fluid
LVs: Lymphatic vessels
mLVs: Meningeal lymphatic vessels
MRI: Magnetic resonance imaging
DCE-MRI: Dynamic contrast-enhanced magnetic resonance imaging
MS: Multiple sclerosis
PD: Parkinson’s disease
TBI: Traumatic brain injury
FLAIR: Fluid-attenuated inversion recovery
VEGFR3: Vascular endothelial growth factor receptor 3
